# Automatisierte Insulinabgabesysteme (AID), physische Aktivität und Sport bei Diabetes mellitus Typ 1: eine gemeinsame Leitlinie der DDG und ÖDG

**DOI:** 10.1007/s00508-025-02663-y

**Published:** 2026-04-30

**Authors:** Othmar Moser, Ulrike Becker, Louisa van den Boom, Christian Brinkmann, Thomas Danne, Elke Fröhlich-Reiterer, Bernhard Gehr, Antonia-Therese Kietaibl, Gerd Köhler, Stephan Kress, Julia K. Mader, Birgit Rami-Merhar, Dominik Pesta, Sabrina Sanfilippo, Ingrid Schütz-Fuhrmann, Harald Sourij, Martin Tauschmann, Ulrike Thurm, Gerlies Treiber, Sabine Hofer

**Affiliations:** 1 Forschungsgruppe für Leistungsphysiologie, Trainingswissenschaften und Trainingstherapie, Institut für Bewegungswissenschaften, Sport und Gesundheit, Universität Graz, Graz, Österreich; 2 Abteilung für Leistungsphysiologie und Metabolismus, BaySpo – Bayreuther Zentrum für Sportwissenschaft, Universität Bayreuth, Bayreuth, Deutschland; 3 Cardiometabolic Trials Unit, Klinische Abteilung für Endokrinologie und Diabetologie, Medizinische Universität Graz, Graz, Österreich; 4 Gesundheitspraxis Bonn, Bonn, Deutschland; 5 Klinik für Kinder- und Jugendmedizin, Helios Klinikum Gifhorn, Gifhorn, Deutschland; 6 Abteilung für Präventive und Rehabilitative Sportmedizin, Deutsche Sporthochschule Köln, Köln, Deutschland; 7 Breakthrough T1D (formerly JDRF), New York, USA; 8 Klinik für Kinder- und Jugendheilkunde Graz, Klinische Abteilung für Allgemeine Pädiatrie, Medizinische Universität Graz, Graz, Österreich; 9 Zentrum für Diabetes- und Stoffwechselerkrankungen, m&i Fachklinik Bad Heilbrunn, Bad Heilbrunn, Deutschland; 10 5. Medizinische Abteilung für Endokrinologie, Rheumatologie und Akutgeriatrie, Klinik Ottakring, Wien, Österreich; 11 Rehabilitationszentrum Aflenz der Pensionsversicherung mit Schwerpunkt Diabetes mellitus und Adipositas, Aflenz, Österreich; 12 Med. Klinik I, Vinzentius-Krankenhaus, Landau, Deutschland; 13 Klinische Abt. für Endokrinologie und Diabetologie, Univ. Klinik für Innere Medizin, Medizinische Universität Graz, Graz, Österreich; 14 Klinische Abteilung für Pädiatrische Pulmologie, Allergologie und Endokrinologie, Univ. Klinik für Kinder- und Jugendheilkunde, Medizinische Universität Wien, Wien, Österreich; 15 Abteilung für Muskel- und Knochenstoffwechsel, Deutsches Zentrum für Luft- und Raumfahrt, Köln, Deutschland; 16 Medizinische Fakultät, Universität zu Köln, Köln, Deutschland; 17 Exzellenzcluster Cellular Stress Responses in Aging-Associated Diseases (CECAD), Köln, Deutschland; 18 3. Medizinische Abteilung mit Stoffwechselerkrankungen und Nephrologie, Karl Landsteiner Institut für Endokrinologie und Stoffwechselerkrankungen, Klinik Hietzing, Wien, Österreich; 19 Universitätsklinik für Pädiatrie 1, Medizinische Universität Innsbruck, Innsbruck, Österreich

**Keywords:** Hypoglykämie, Glukosedynamik, Trainingsanpassung, Stoffwechselregulation, Belastungsstreuerung, Hypoglycemia, Glucose-dynamics, Training-adaptation, Metabolic-regulation, Exercise-management

## Abstract

In den letzten Jahren hat die bessere Verfügbarkeit und Nutzung automatisierter Insulinabgabesysteme (AID) gezeigt, dass Menschen mit AID-Behandlung in höherem Ausmaß glykämische Therapieziele erreichen und dass dies mit einer Verbesserung der Lebensqualität einhergeht. Regelmäßige physische Aktivität und Sport sind grundlegende Bestandteile der Diabetesbehandlung bei Menschen mit Diabetes mellitus Typ 1 und verbessern neben der glykämischen Einstellung und der allgemeinen Gesundheit auch die Lebensqualität. Trotz der klaren Evidenz für die positiven Effekte von physischer Aktivität und Sport stellen Glukoseschwankungen während dieser Aktivitäten eine Herausforderung für Menschen mit Diabetes und ihre Therapie dar. Die Zunahme an Evidenz über die letzten Jahre mit vermehrt klinischen Studien und Übersichtsarbeiten zu verschiedenen AID-Systemen bei physischer Aktivität und Sport führte zur Erstellung eines gemeinsamen Positionspapiers zweier großer Diabetesgesellschaften, der Europäischen Diabetes Gesellschaft (EASD) und der Internationalen Gesellschaft für pädiatrischen und adoleszenten Diabetes (ISPAD). Dieses Positionspapier fasst die aktuelle Evidenz zu AID-Systemen zusammen und bietet praxisnahe Empfehlungen für das Management von physischer Aktivität und Sport bei Kindern, Jugendlichen und Erwachsenen mit Diabetes mellitus Typ 1 unter Nutzung von AID-Systemen. Diese Arbeit bietet die Grundlage für die hier vorliegende deutschsprachige gemeinsame Leitlinie der Deutschen Diabetes Gesellschaft (DDG) und der Österreichischen Diabetes Gesellschaft (ÖDG) zum Thema AID, physische Aktivität und Sport bei Diabetes mellitus Typ 1. In dieser Leitlinie werden alle in Deutschland und Österreich verfügbaren AID-Systeme vorgestellt und systemspezifische Empfehlungen für deren Anwendung bei physischer Aktivität und Sport gegeben. Darüber hinaus werden unterschiedliche Glukosereaktionen für physische Aktivität und Sport besprochen und differenzierte Therapieoptionen aufgezeigt, um Glukoseschwankungen zu vermeiden und den gewünschten Glukosezielbereich zu erreichen.

## Abstract (en)

The increased availability and use of automated insulin delivery (AID) systems in recent years have shown that individuals using AID systems can more easily and consistently achieve glycemic targets and that this is associated with improvements in quality of life. Alongside this technological progress, regular physical activity and exercise remain cornerstones of diabetes management for individuals with type 1 diabetes, owing to the numerous well-documented health benefits of an active lifestyle. Despite the clear evidence supporting the positive effects of physical activity and exercise, glucose fluctuations frequently occur around physical activity and exercise, posing challenges for AID systems and simultaneously deterring people with type 1 diabetes from maintaining a physically active lifestyle. Given the growing number of clinical trials and reviews on AID systems in the context of physical activity and exercise, there is a clear need to consolidate this knowledge and develop joint guidelines by the German Diabetes Association (DDG) and the Austrian Diabetes Association (ÖDG). This German-language guideline is based on the current position statements of the European Association for the Study of Diabetes (EASD) and the International Society for Pediatric and Adolescent Diabetes (ISPAD). They summarize the evidence on AID systems and provide practical recommendations for the management of physical activity and exercise in children, adolescents and adults with type 1 diabetes using AID systems. Within the guidelines we detail each AID system available in Germany and Austria and give specific guidance for their use around physical activity and exercise. Furthermore, we discuss glycemic responses to physical activity and exercise and outline therapeutic strategies to maintain glycemia within target ranges.

## Diabetes mellitus Typ 1 – Bewegung, Sport und AID

Die neue gemeinsame Leitlinie „Automatisierte Insulinabgabesysteme (AID), physische Aktivität und Sport bei Diabetes mellitus Typ 1: eine gemeinsame Leitlinie der DDG und ÖDG“ stellt alle derzeit in Deutschland und Österreich verfügbaren AID-Systeme vor und beschreibt Empfehlungen für deren Anwendung bei physischer Aktivität und Sport. Zusätzlich werden unterschiedliche Glukosereaktionen für physische Aktivität und Sport besprochen und differenzierte Therapieoptionen erläutert, um Glukoseschwankungen zu vermeiden und den gewünschten Glukosezielbereich während und nach der physischen Aktivität zu erreichen.

## Einleitung

**Abb. 1:**
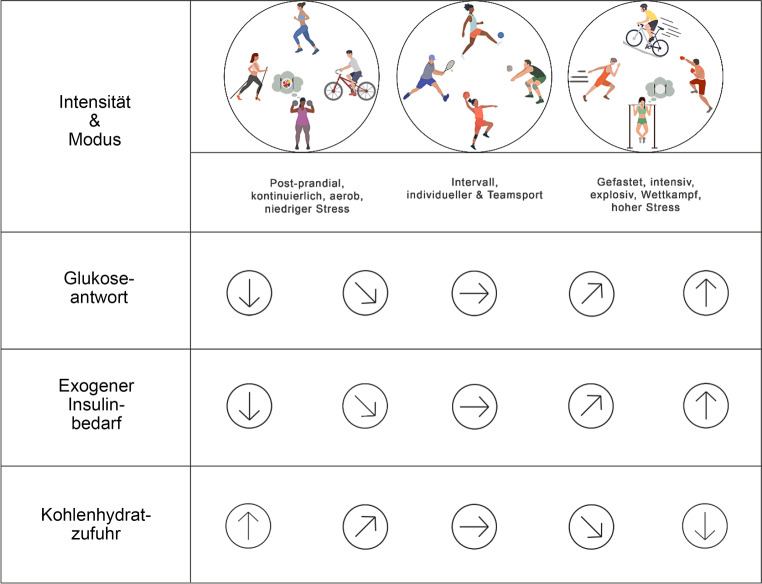
Allgemeiner Überblick über die Glukosetrends und den Bedarf an exogenem Insulin und Kohlenhydraten als Reaktion auf körperliche Aktivität bei Menschen mit T1DM

Regelmäßige physische Aktivität und Sport mit moderater bis hoher Intensität können wesentlich zum erfolgreichen Management des Diabetes mellitus Typ 1 (T1DM) beitragen [1, 2]. Während frühere und bestehende Leitlinien Empfehlungen zur Glukosekontrolle während physischer Aktivität und Sport auf Grundlage glykämischer Trends und kontinuierlicher Glukosemessung (CGM) geben [1–5], liegen bislang für aktuell kommerziell verfügbare automatisierte Insulinabgabesysteme (AID) nur begrenzt Handlungsempfehlungen im Kontext physischer Aktivität und Sport vor [6–9]. Unterschiedliche Sportarten beeinflussen die Glukoseschwankungen in unterschiedlichem Ausmaß (Abb. 1). Die gemeinsame Leitlinie zu AID, physischer Aktivität und Sport der Europäischen Diabetes Gesellschaft (EASD) und Internationalen Gesellschaft für pädiatrischen und adoleszenten Diabetes (ISPAD) bilden die Grundlage für die nachfolgend beschriebenen allgemeinen Prinzipien bei der Nutzung von AID-Systemen im Rahmen von physischer Aktivität und Sport [10, 11].

Nutzer:innen von AID-Systemen berichten regelmäßig über Herausforderungen in Bezug auf Mahlzeiten [12, 13], physische Aktivität sowie geplante und ungeplante (spontane) sportliche Betätigung [9]. Zusätzlich bestehen verschiedene Hürden vor physischer Aktivität – etwa die Angst vor Hypoglykämien –, die mit einem erhöhten Risiko für „Diabetes-Distress“ assoziiert sein können [14]. Derzeit sollten Nutzer:innen von AID-Systemen Mahlzeiten, physische Aktivität und Sport im Voraus planen und dem System dies durch manuelle Eingabe ankündigen. Insbesondere rasche Änderungen der Glukose z. B. nach Mahlzeiten oder bei Sport und Bewegung [15–20] sowie physiologisch und technologisch bedingte Unterschiede zwischen Blutglukosewerten und Messwerten aus der interstitiellen Flüssigkeit stellen diese technischen Systeme vor Herausforderungen.

In dieser gemeinsamen Leitlinie der DDG und ÖDG wird die derzeitige Evidenzlage zur Nutzung kommerziell verfügbarer AID-Systeme im Kontext physischer Aktivität und Sport dargestellt. Darüber hinaus werden konkrete Handlungsempfehlungen für das Management eines aktiven Lebensstils bei Kindern, Jugendlichen und Erwachsenen mit T1DM gegeben. Ergänzende Informationen zu neuen Entwicklungen im Bereich der AID-Technologie können im Positionspapier der EASD und ISPAD nachgelesen werden, die als Basis und Grundlage dieser deutschsprachigen Leitlinie dienen [10, 11]. Unsere Leitlinie richtet sich sowohl an das medizinische Fachpersonal als auch an Menschen mit T1DM und verfolgt das Ziel, effektive Strategien zum besseren Glukosemanagement im Zusammenhang mit geplanter und ungeplanter (spontaner) physischer Aktivität und Sport bereitzustellen. Die hier vorliegende Leitlinie bietet daher einen umfassenden Überblick über physische Aktivität, Sport und kommerziell verfügbare AID-Systeme und soll als Grundlage für ein sicheres und effektives Bewegungsmanagement dienen, um von den zahlreichen positiven Effekten eines aktiven Lebensstils profitieren zu können. Innerhalb dieser Leitlinie wird physische Aktivität als „jegliche Körperbewegung bezeichnet, die mit einer Muskelkontraktion verbunden ist und bei der der Energieverbrauch höher als im Ruhezustand ist“ [21]. „Beim Sport ist es das Ziel, die körperliche Fitness in einem oder mehreren Bereichen zu verbessern oder zu erhalten.“ [22]

## Methodik zur Konsensfindung der Leitliniengruppe

Die Mitglieder der Autor:innengruppe wurden am 03.02.2025 durch den korrespondierenden Autor auf Grundlage ihrer Publikationstätigkeit und/oder klinischen Expertise im Bereich AID-Systeme, physische Aktivität und Sport ausgewählt und am 06.03.2025 durch die DDG und ÖDG bestätigt. Nach ersten Abstimmungen im engeren Autor:innenteam erstellten der Erstautor und die Letztautorin einen Entwurf, der am 19.05.2025 der gesamten Gruppe zur Diskussion vorlag. Zur strukturierten Konsensfindung fand am 26.05.2025 eine Online-Sitzung statt. Nach Einarbeitung der Rückmeldungen wurde die überarbeitete Version am 29.06.2025 zur Begutachtung bei der DDG und ÖDG eingereicht. Nach deren Stellungnahme wurde die finale Version am 02.10.2025 der Autor:innengruppe zur Freigabe vorgelegt und anschließend von den Leitlinienkommissionen sowie den Vorständen beider Fachgesellschaften anerkannt.

## Datenquellen, Literaturrecherche und Studienauswahl

Als methodische Grundlage wurde das Positionspapier der EASD und ISPAD zu AID, physischer Aktivität und Sport gewählt [10, 11]. Die Stärke der Empfehlungen basierend auf dem Evidenzniveau in dieser Leitlinie wird gemäß einer A‑ bis D‑Kategorisierung dargestellt. Die Empfehlungen, die als „Konsens D“ bezeichnet werden, basieren auf klinischer Erfahrung und der Einschätzung von Expert:innen.

## Konsensbasierte Empfehlungen

Die Leitliniengruppe empfiehlt 5 zentrale Strategien für das Management von physischer Aktivität, Sport und T1DM bei Anwendung von AID-Technologien, unabhängig vom jeweiligen Hersteller der AID-Systeme (Tab. 1). Die Autor:innen sind der Meinung, dass diese Empfehlungen für den Großteil, jedoch nicht für alle Arten physischer Aktivität und Sport anwendbar sind (Konsens D).

**Tab. 1:** Allgemeine Überlegungen zu physischer Aktivität und Sport bei der Verwendung von automatisierten Insulindosiersystemen (*AID*)

Nr.	Empfehlung bei physischer Aktivität und Sport bei AID (Evidenzstärke)
1	Bei geplanter physischer Aktivität und Sport mit zu erwartendem Glukoseabfall wird empfohlen, den Glukosezielwert 1–2 h vor Beginn der Aktivität zu erhöhen [23–26] (A). Ist hingegen ein Glukoseanstieg während der Aktivität zu erwarten, kann das persönliche Glukoseziel unverändert beibehalten oder gegebenenfalls reduziert werden (Konsens D)
2	Bei geplanter physischer Aktivität und Sport innerhalb von 2 h nach einer kohlenhydratreichen Mahlzeit kann die prandiale Insulindosis reduziert werden, beispielsweise um 25–33 %, wenn ein Glukoseabfall während der Aktivität zu erwarten ist [23, 24] (B). Zuvor kann der Glukosezielwert erhöht werden, um eine Erhöhung der automatisierten Insulinabgabe zu vermeiden [24, 27] (D)
3	CGM-Werte und Trendpfeile sollten sorgfältig beobachtet werden. Bei Sensor-Glukosewerten < 126 mg/dl (< 7,0 mmol/l) während der Aktivität können kleine Mengen schnell wirksamer Kohlenhydrate (3–20 g) zugeführt werden [27]. Zu viele Kohlenhydrate können zu einem Glukoseanstieg und daraus resultierend gegebenenfalls zu einer Erhöhung der automatisierten Insulinabgabe führen und damit das Risiko einer Hypoglykämie während oder unmittelbar nach der Aktivität erhöhen (D)
4	Bei zu erwartendem Glukoseabfall bei ungeplanter physischer Aktivität und Sport kann unmittelbar zu Beginn der körperlichen Belastung das persönliche Glukoseziel erhöht werden. Zusätzlich können 10–20 g schnell wirksame Kohlenhydrate aufgenommen werden, wenn der Sensorglukosewert < 126 mg/dl (< 7,0 mmol/l) liegt [7, 8]. Wird hingegen ein Glukoseanstieg erwartet, kann der persönliche Glukosezielwert beibehalten oder gegebenenfalls reduziert werden (D)
5	Wenn möglich, sollte zum Zeitpunkt physischer Aktivität wenig *Insulin On Board* (IOB)^1^ vorliegen, wie dies z. B. vor Mahlzeiten oder im nüchternen Zustand der Fall ist [28, 29] (B). Bei postprandial erhöhten Glukosewerten kann eine Aktivität mit niedriger Intensität empfohlen werden, da diese typischerweise zu einer Normalisierung des Glukosespiegels beiträgt/führt [30, 31] (C). Wenn die Glukosekonzentration über längere Zeit unvorhergesehen > 270 mg/dl (> 15,0 mmol/l) liegt, entscheidet die Blut- oder Gewebsketonkonzentration (> 1,5 mmol/l), ob physische Aktivität und Sport vermieden werden sollten [4, 33] (D)

^1^ Insulinspiegel im Blut

## Allgemeine Empfehlungen zu physischer Aktivität und Sport bei Verwendung automatisierter Insulinabgabesysteme (AID)

Aufgrund der Vielfalt an Bewegungsformen mit unterschiedlichen Belastungsmustern sind differenzierte Empfehlungen zu körperlicher Aktivität und Sport notwendig. In Abhängigkeit der Sportarten (Ausdauersport, gemischte Sportarten und Kraftsport), Stress und je nachdem, ob diese geplant oder ungeplant erfolgen, variieren die Behandlungsempfehlungen [7–9].

Die meisten AID-Systeme bieten die Möglichkeit, den Glukosezielwert individuell und für einen vorprogrammierten Zeitraum zu ändern, um vor, während und nach der Aktivität eine möglichst hohe Zeit im Zielbereich (70–180 mg/dl; 3,9–10,0 mmol/l) zu erreichen. Je nach verwendetem AID-System wird die Funktion zur Erhöhung des Glukosezielwertes unterschiedlich bezeichnet. Im Sinne einer einheitlichen Terminologie wird im Folgenden in dieser Leitlinie der Begriff „erhöhter Glukosezielwert“ verwendet.

Eine zentrale Herausforderung bei der Nutzung von AID-Systemen im Rahmen physischer Aktivität und Sport besteht darin, eine vor Aktivitätsbeginn unerwünschte Erhöhung der automatisierten Insulinzufuhr zu vermeiden – insbesondere in Situationen mit steigenden oder bereits erhöhten Sensor-Glukosewerten infolge eines kohlenhydrathaltigen Snacks oder einer reduzierten prandialen Insulindosis. Durch frühzeitige Erhöhung des Glukosezielwertes bis zu 120 min vor Beginn der Aktivität kann diese automatisierte Insulinzufuhr gedrosselt werden.

Grundsätzlich kann das AID-System auch während der Aktivität getragen und im Automodus belassen werden. Sollten wiederholt aktivitätsinduzierte Hypoglykämien auftreten, kann in seltenen Fällen und in Rücksprache mit dem Diabetesteam das System vor Beginn der Aktivität in den manuellen Modus gestellt werden, alternativ kann auch ein vollständiges Abkoppeln des AID-Systems erforderlich sein. Im Falle eines Ablegens/Abkoppelns des AID-Systems ist es relevant, den Automodus aktiv zu beenden, um zu verhindern, dass Insulin vom System abgegeben und als IOB berechnet wird, das nie im Körper angekommen ist. In Einzelfällen kann das Sportregime auch Insulininjektionen mittels Pen erfordern.

Sinkt der Glukosewert während der Aktivität unter 126 mg/dl (< 7,0 mmol/l) trotz aktivierten erhöhten Glukosezielwerts, kann auf Grundlage des Sensorglukosetrendpfeils eine kleine Menge schnell wirksamer Kohlenhydrate zugeführt werden [8, 27, 34–36] (C). Die Basisempfehlungen dazu lauten:

- 3–6 g KH bei horizontalem Sensorglukosetrendpfeil,
- 6–9 g KH bei leicht fallendem Sensorglukosetrendpfeil,
- 9–12 g KH bei deutlich fallendem Sensorglukosetrendpfeil,
- 12–20 g KH bei 2 bis 3 fallenden Sensorglukosetrendpfeilen.

Empfohlen wird zudem eine erneute Kontrolle des Sensorglukosewertes ca. 20–30 min nach der Kohlenhydrataufnahme und ggf. eine Wiederholung der Maßnahme [4] (Konsens D). Grundsätzlich beziehen sich die größeren Kohlenhydratmengen auf Erwachsene; für Kinder und Jugendliche sind eher niedrigere Kohlenhydratmengen zu erwarten (Konsens D).

Nach der Aktivität kann der Sensorglukosezielwert wieder auf den regulären Glukosezielwert zurückgesetzt werden oder für eine gewisse Zeit erhöht bleiben, wenn mit sinkenden Glukosewerten nach der Aktivität zu rechnen ist [27, 37–39] (C).

## AID-systemspezifische Empfehlungen

Die systemspezifischen Empfehlungen zu AID-Systemen für Deutschland und Österreich sind in alphabetischer Reihenfolge nach Herstellern aufgeführt.

## CamDiab mylife CamAPS FX

Das mylife CamAPS FX-System (CamDiab Ltd, Cambridge, Cambridgeshire, Vereinigtes Königreich) ermöglicht die individuelle Einstellung eines persönlichen Glukosezielwertes im Bereich von 80–198 mg/dl (4,4–11,0 mmol/l), wobei der Standardglukosezielwert bei 104 mg/dl (5,8 mmol/l) liegt. Die Insulinabgabe erfolgt im Automodus mittels algorithmusgesteuerter Insulinmodulation; manuelle Korrekturbolusgaben sind im Automodus möglich, jedoch nicht empfohlen. Im System zählt jede über den Bolusrechner verabreichte Insulindosis (unabhängig davon, ob sie korrektur- oder mahlzeitenbezogen ist) zum IOB, das als „aktives Insulin“ angezeigt wird. Die vom System angezeigte Wirkdauer des „aktiven Insulins“ kann vom Benutzer auf 2–8 h eingestellt werden, wobei die vom Algorithmus tatsächlich berücksichtigte Wirkdauer adaptiv erlernt und automatisch angepasst wird. Die basale algorithmusgesteuerte Insulinzufuhr wird nicht zur IOB gerechnet, und die eingestellte Insulinwirkdauer hat keinen Einfluss auf die algorithmusgesteuerte Insulinabgabe. Eine realistische Darstellung des IOB kann über die Querformatansicht oder über die Infoschaltfläche auf dem Smartphone eingesehen werden, in der sowohl die letzten Bolusgaben als auch das pharmakokinetische Profil der algorithmusgesteuerten Insulinzufuhr grafisch dargestellt werden.

Das System verfügt über 2 zusätzliche Modi:

- *„Ease-off“*-Modus: reduziert die Insulinabgabe, hebt den Glukosezielwert an und pausiert die Insulinzufuhr spätestens bei Glukosewerten < 126 mg/dl (< 7,0 mmol/l),
- *Boost*-Modus: erhöht die Reaktionsintensität des Algorithmus auf erhöhte Glukosewerte, während der Glukosezielwert unverändert bleibt.

## Evidenz zum Glukosemanagement bei physischer Aktivität und Sport mit dem mylife CamAPS FX-System

Studien bei Kindern und Jugendlichen mit T1DM zeigen, dass die Aktivierung des *„Ease-off“*-Modus im Rahmen physischer Aktivität zu einer sicheren Glukosekontrolle führt [27, 40]. So konnte durch Erhöhung des Glukosezielwertes auf 150 mg/dl (8,3 mmol/l) und gleichzeitiger Aktivierung des *„Ease-off“*-Modus 2 h vor einem maximalen kardiopulmonalen Belastungstest eine stabile Glukosekonzentration im Verlauf erreicht werden [40]. Auch in einer Skifreizeitstudie bei Kindern und Jugendlichen konnten durch den Start des *„Ease-off“*-Modus 2 h vor dem Sport Hypoglykämien vermieden werden [27].

## Empfehlungen zum Glukosemanagement bei physischer Aktivität und Sport mit dem mylife CamAPS FX-System

Zur Reduktion des Hypoglykämierisikos während physischer Aktivität und Sport wird empfohlen, den *„Ease-off“*-Modus zu aktivieren und/oder den Glukosezielwert 1–2 h vor Beginn der Aktivität zu erhöhen [27, 40] (C). Dies ist besonders relevant bei hohem IOB oder aerober Aktivität [41] (Konsens D). Bei Sportarten, die einen Glukoseabfall erwarten lassen, kann der Glukosezielwert bis zu 2 h vor Beginn der Aktivität auf ≥ 150 mg/dl (≥ 8,3 mmol/l) eingestellt werden. Wir empfehlen, dass Nutzer:innen bei Verwendung des *„Ease-off“*-Modus (erhöhter Zielwert) oder *Boost*-Modus (erniedrigter Zielwert) einen individuellen Zielwert für physische Aktivität definieren, um das gewünschte Glukoseniveau bestmöglich zu erreichen [40] (Konsens D). Bei erwartetem Glukoseanstieg (z. B. bei hochintensivem Intervalltraining im nüchternen Zustand oder in Wettkampfsituationen) kann der *Boost*-Modus aktiviert werden [9, 28] (Konsens D). Beide Modi lassen sich – sofern gewünscht – zeitgesteuert vorkonfigurieren, sodass sie automatisch zu definierten Zeiten starten und enden. Dies kann sinnvoll sein, wenn eine Aktivität zu fix geplanter Zeit stattfindet, oder im Falle von Aktivität kurz nach dem Erwachen.

Bei ungeplanter Aktivität mit niedriger bis moderater Intensität, bei der ein Glukoseabfall erwartet wird und die aktuellen Werte bereits im Zielbereich liegen, sollten der *„Ease-off“*-Modus und/oder ein erhöhter Glukosezielwert sofort zu Beginn der ungeplanten Aktivität oder so früh wie möglich aktiviert werden. Zusätzlich wird eine Aufnahme von 10–20 g schnell wirksamer Kohlenhydrate zu Beginn der Aktivität empfohlen, jedenfalls, wenn ein Glukosewert unter 126 mg/dl (< 7,0 mmol/l) vorliegt [8, 42] (Konsens D). Diese Mahlzeit sollte in das System über die Snack-Funktion als Hypoglykämiemahlzeit eingegeben werden, um keine Reaktion des Algorithmus zu provozieren [27, 40] (C). Wie bei jeder längeren körperlichen Aktivität kann abhängig von Glukosetrends oder Leistungsanforderungen eine zusätzliche Kohlenhydratzufuhr notwendig sein [43]. Erwartet man hingegen, basierend auf Erfahrung, einen Glukoseanstieg [25], kann der *Boost*-Modus mit dem regulären oder gegebenenfalls reduzierten Glukosezielwert aktiviert werden. Dies sollte jedoch erst unmittelbar zu Beginn der Aktivität geschehen, um aktivitätsbedingte Hyperglykämien und AID-induzierte Hypoglykämien zu vermeiden. Ein zu frühes Aktivieren des *Boost*-Modus könnte hingegen das Risiko einer Hypoglykämie erhöhen (Konsens D). Jedenfalls ist diese Empfehlung erfahrenen Sportler:innen vorbehalten, und wir empfehlen, bei Kindern und Jugendlichen sowie unerfahrenen Sportler:innen dieses Vorgehen nur in enger Absprache mit ihrem Diabetesteam zu testen. Da der *„Ease-off“*- und *Boost*-Modus nur begrenzt in die lernbasierte Steuerung des Algorithmus einfließen, können sie insbesondere für Personen empfohlen werden, die unregelmäßig körperlich aktiv sind (Konsens D). Bei regelmäßig wiederkehrender Aktivität (z. B. montags, mittwochs, freitags und sonntags jeweils um ~17:00 Uhr) kann ein individueller Zielwert in Abhängigkeit von Uhrzeit, Zeitpunkt der letzten Mahlzeit bzw. IOB und Art der Aktivität sinnvoll sein (Konsens D).

**Abb. 2:**
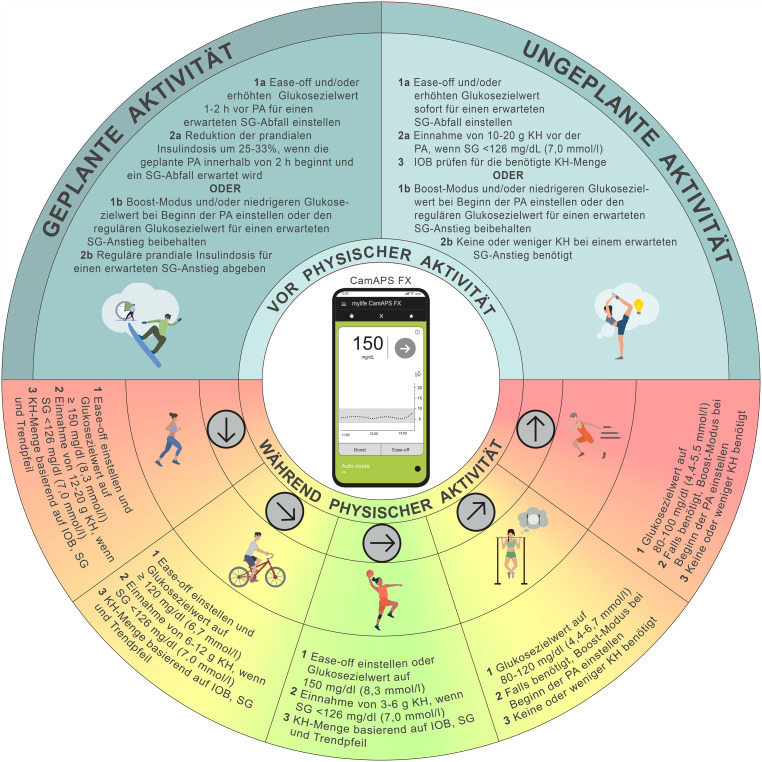
Empfehlungen zur Anwendung des mylife CamAPS FX-Systems zur Steuerung der Glukosewerte während physischer Aktivität und Sport (*PA*): Unter bestimmten Umständen – wie etwa in Wettkampfsituationen, beim Schwimmen, Tauchen oder bei Kontaktsportarten – kann eine temporäre Unterbrechung der Insulinzufuhr mit oder ohne Abkoppelung des Systems über einen längeren Zeitraum (bis zu 120 min) erforderlich sein. Für die meisten Aktivitäten wird dies jedoch nicht generell empfohlen. *SG* Sensor-Glukosewert, *KH* Kohlenhydrate, *IOB* Insulin On Board

Bei Nutzung des mylife CamAPS FX-Systems empfehlen wir, basierend auf Erfahrung, den Glukosezielwert individuell an die erwartete glykämische Reaktion auf physische Aktivität anzupassen (Konsens D). Alle empfohlenen Anpassungen zu Moduswahl und Glukosezielwerten sind in Abb. 2 dargestellt.

## Diabeloop Generation 1 (DBLG1)

Das DBLG1-System (Diabeloop SA, Grenoble, Frankreich) der ersten Generation verfügt über einen standardmäßig voreingestellten Glukosezielwert von 110 mg/dl (6,1 mmol/l), wobei dieser individuell im Bereich von 100–130 mg/dl (5,6–7,2 mmol/l) angepasst werden kann. Die Schwelle für niedrige Glukosewerte, deren Erreichen der Algorithmus auf jeden Fall zu vermeiden versucht, kann zwischen 60 und 85 mg/dl (3,3–4,7 mmol/l) eingestellt werden. Die Grenze für Hyperglykämie, bei der der Algorithmus reagiert, liegt bei 180 mg/dl (10,0 mmol/l). Die Insulinabgabe kann hinsichtlich ihrer Intensität im Bereich von 59–147 % der üblichen Basalrate reguliert werden, wenn die Glukosewerte zwischen 70 und 180 mg/dl (3,9–10,0 mmol/l) liegen. Bei Glukosewerten über 180 mg/dl (10,0 mmol/l) kann die automatisierte Korrekturbolusgabe auf einen Bereich von 43–186 % der standardisierten automatisierten Korrekturbolusdosis eingestellt werden. Zudem lässt sich die prandiale Insulinzufuhr für Frühstück, Mittag- und Abendessen im Bereich von 50–200 % anpassen. Diese Funktion ermöglicht eine Anpassung der Mahlzeiteninsulindosis in Bezug auf nachfolgende physische Aktivität und Sport; dies sollte jedoch stets individualisiert und in Absprache mit dem behandelnden medizinischen Fachpersonal erfolgen. Im System wird das aktive Insulin (IOB, „Aktives Insulin“) in der Benutzeroberfläche angezeigt. Es umfasst das durch den Algorithmus gesteuerte Insulin und berücksichtigt sowohl Basalraten als auch Bolusgaben, wie sie von der Insulinpumpe verifiziert wurden.

## Evidenz zum Glukosemanagement bei physischer Aktivität und Sport mit dem DBLG1-System

In einer Post-hoc-Analyse wurde bei 56 Erwachsenen mit T1DM, die das DBLG1-System über 12 Wochen nutzten, die Glykämie an Tagen mit und ohne körperliche Aktivität verglichen [39]. Die Aktivität wurde mindestens 30 min vorher im System angegeben; bei Bedarf empfahl das System Kohlenhydrate zur Hypoglykämieprävention. Die Zeit unter dem Zielbereich (< 54 mg/dl/< 3,0 mmol/l) unterschied sich nicht signifikant, unabhängig von Dauer und Intensität der Aktivität. Die Zeit im Zielbereich (70–180 mg/dl; 3,9–10,0 mmol/l) war jedoch an Aktivitätstagen geringer. Eine weitere Studie zeigte, dass das DBLG1-System dem Open-Loop-Modus überlegen war, wenn der Modus „Körperliche Aktivität“ 30 min vor Beginn aktiviert wurde, sowohl hinsichtlich der Zeit im Zielbereich als auch über dem Zielbereich (> 180 mg/dl; > 10,0 mmol/l) [40].

## Empfehlungen zum Glukosemanagement bei physischer Aktivität und Sport mit dem DBLG1-System

**Abb. 3:**
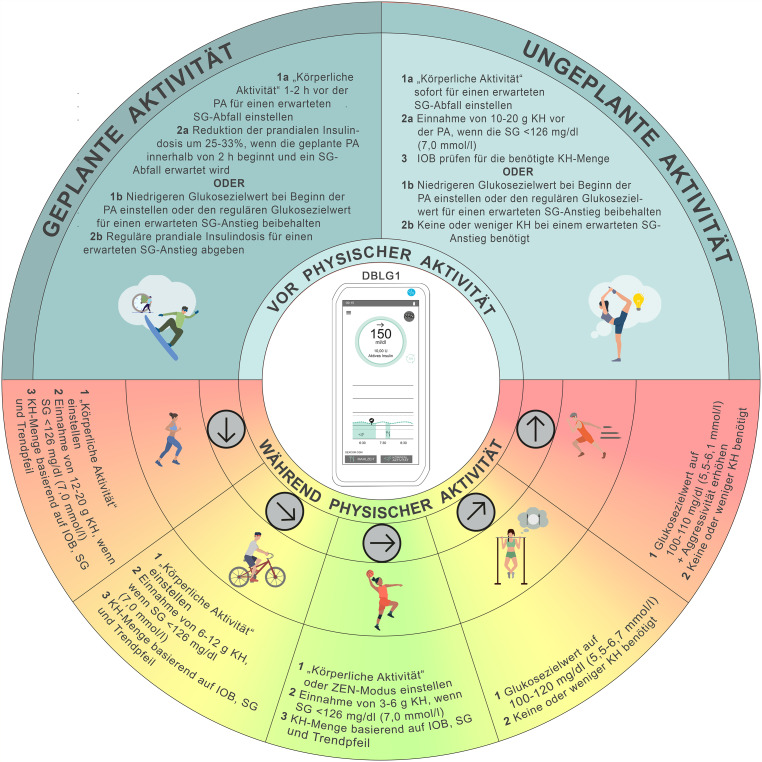
Empfehlungen zur Anwendung des DBLG1-Systems zur Steuerung der Glukosewerte während physischer Aktivität und Sport (*PA*): Eine Unterbrechung der Insulinzufuhr mit oder ohne Abkoppelung über einen Zeitraum von bis zu 120 min kann bei bestimmten Aktivitäten (z. B. Schwimmen, Tauchen, Kontaktsport oder in Wettkampfsituationen) erforderlich sein. Für die Mehrheit der Aktivitäten wird dies jedoch nicht empfohlen. *SG* Sensor-Glukosewert, *KH* Kohlenhydrate, *IOB* Insulin On Board

Der Modus für „*körperliche Aktivität“* kann eingesetzt werden, um das Risiko für Hypoglykämien während und nach der physischen Aktivität und Sport zu senken (Abb. 3). In diesem Modus werden das Glukoseziel und die Hypoglykämieschwelle um 70 mg/dl (3,9 mmol/l) erhöht, was zu einer Reduktion der Insulinabgabe führt. Es kann die Belastungsintensität als niedrig, moderat oder intensiv angegeben werden, ebenso wie die geplante Dauer der körperlichen Aktivität. Beide Parameter beeinflussen über eine Matrix die Modulation der Basalrate, der Korrekturbolusabgaben und/oder der Mahlzeiteninsulinzufuhr. Eine weitere Funktion des DBLG1-Systems ist der „ZEN“-Modus, der den Glukosezielwert für eine Dauer von 1–8 h um 10–40 mg/dl (0,6–2,2 mmol/l) erhöht [9] (D). Es wird empfohlen, den Modus „*körperliche Aktivität“* mindestens 30 min vor Bewegungsbeginn zu aktivieren, da das System eine definierte Kohlenhydratmenge zur Hypoglykämieprophylaxe vorschlägt [44] (C). Ein noch früheres Aktivieren (z. B. 1–2 h vorher) kann das Hypoglykämierisiko zusätzlich senken [25, 27] (D). Werden die physische Aktivität und Sport mehr als 1 h vor Beginn angekündigt, wird der Zielwert um 70 mg/dl (3,9 mmol/l) erhöht, und das System strebt einen Glukoseanstieg vor Aktivitätsbeginn an. Liegt der Glukosewert 15 min vor der Aktivität < 160 mg/dl (< 8,9 mmol/l), wird vom System eine spezifische Kohlenhydratzufuhr empfohlen. Erfolgt die Ankündigung weniger als 1 h vor der physischen Aktivität und Sport, gibt das System lediglich bei Werten unter 160 mg/dl (< 8,9 mmol/l) eine Kohlenhydratempfehlung ab. Zusätzlich reduziert das DBLG1-System nach Aktivierung des physischen Aktivität-Modus die Basalrate automatisch über einen Zeitraum von 16 h, um das Langzeitrisiko für Hypoglykämien durch erhöhte Insulinsensitivität zu minimieren. Der Modus „*körperliche Aktivität“* erlaubt es außerdem, die Aktivität zu benennen und zu speichern (z. B. „Fußball“) sowie Dauer und Intensität zu hinterlegen (niedrig, moderat, intensiv). Im neuen Update wurde der Algorithmus für *„körperliche Aktivität“* überarbeitet; nun kann aus einer Liste von Sportarten mit jeweils hinterlegter Intensitätsangabe („aerob“, „anaerob“ oder „gemischt“) gewählt werden. Bei Nutzung des DBLG1-Systems empfehlen wir, basierend auf Erfahrung, den Glukosezielwert individuell an die erwartete glykämische Reaktion auf physische Aktivität anzupassen (Konsens D). Die Abb. 3 zeigt Handlungsempfehlungen für das Glukosemanagement bei PA unter Verwendung des DBLG1-Systems.

## Insulet Omnipod 5

Das Omnipod 5-System (Insulet Corporation, Acton, Massachusetts, USA) ist ein schlauchloses AID-System [9, 45], das mithilfe der sog. *SmartAdjust*-Technologie Glukosewerte bis zu 60 min im Voraus prognostiziert und die basale Insulinzufuhr alle 5 min dynamisch anpasst. Die *SmartAdjust*-Funktion steuert Glukosewerte im Bereich von 110–150 mg/dl (6,1–8,3 mmol/l); der Zielwert kann von Nutzer:in, Betreuungsperson oder medizinischem Fachpersonal festgelegt und tageszeitabhängig variiert werden. Die adaptive Basalrate stellt einen zentralen Bestandteil der *SmartAdjust*-Technologie dar. Diese Rate wird bei jedem Pod-Wechsel aktualisiert und basiert auf der historischen Insulinabgabe, die sowohl Basal als auch Bolusinsulin umfasst. Die Berechnung erfolgt mittels eines abklingend gewichteten Mittelwerts, wobei die letzten 4 bis 5 Pods (entspricht 12–15 Anwendungstage) den stärksten Einfluss auf die adaptive Basalrate haben. Zudem handelt es sich beim Omnipod 5 um eine wasserdichte Patchpumpe, die insbesondere bei wasserbasierten Aktivitäten eine erhöhte Flexibilität bietet [46].

Das IOB setzt sich im Omnipod 5 aus 3 Komponenten zusammen:

1. Korrektur-IOB (Restinsulin aus früheren Korrekturboli),
2. Mahlzeiten-IOB (Restinsulin aus Mahlzeitenboli) sowie
3. Software-IOB (alle vom System abgegebenen Insulindosen).

Das IOB wird maßgeblich durch die Einstellung der „Wirkdauer des Insulins“ bestimmt, die zwischen 2 und 6 h wählbar ist. Die Funktion „Gegenläufige Korrektur“ zieht vorhandenes IOB von der Bolusberechnung ab, wenn der aktuelle Glukosewert unter dem Zielwert liegt [45].

## Evidenz zum Glukosemanagement bei physischer Aktivität und Sport mit dem Omnipod 5-System

In der Zulassungsstudie zum Omnipod 5-System wurde eine Trainingsstudie mit 59 Erwachsenen mit T1DM durchgeführt, in welcher gezeigt wurde, dass sowohl 30 min als auch 60 min vor dem Sport die Aktivierung der *Aktivität-Funktion* die automatisierte Insulinabgabe reduziert und ein höherer Glukosewert im Sport erreicht wird [47].

## Empfehlungen zum Glukosemanagement bei physischer Aktivität und Sport mit dem Omnipod 5-System

**Abb. 4:**
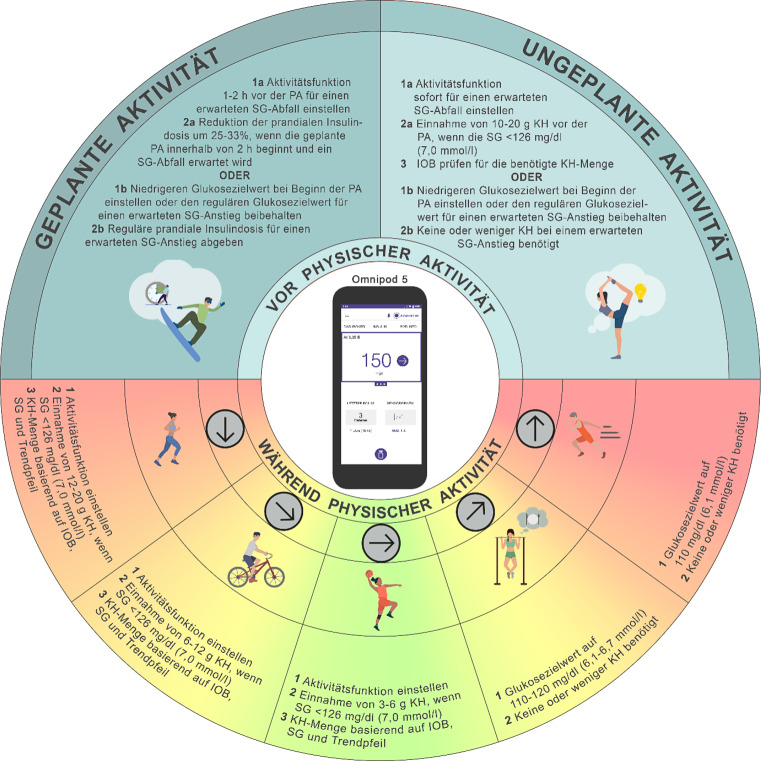
Empfehlungen zur Anwendung des Omnipod 5-Systems zur Steuerung der Glukosewerte während physischer Aktivität und Sport (*PA*): Eine Unterbrechung der Insulinzufuhr mit oder ohne Abkoppelung über einen Zeitraum von bis zu 120 min kann bei bestimmten Aktivitäten (z. B. Schwimmen, Tauchen, Kontaktsport oder in Wettkampfsituationen) erforderlich sein. Für die Mehrheit der Aktivitäten wird dies jedoch nicht empfohlen. *SG* Sensor-Glukosewert, *KH* Kohlenhydrate, *IOB* Insulin On Board

Für den Einsatz bei physischer Aktivität und Sport beträgt der erhöhte Glukosezielwert im Omnipod 5-System 150 mg/dl (8,3 mmol/l). Dieser Zielwert führt zu einer Reduktion der automatisierten Insulinzufuhr und kann für eine Dauer von 1–24 h eingestellt werden [7]. Bei Aktivitäten mit erhöhtem Hypoglykämierisiko wird empfohlen, die Funktion „*Aktivität*“ 1–2 h vor Beginn bis zum Ende der Aktivität zu aktivieren (Abb. 4). Wenn standardmäßig Zielwerte von 120, 130, 140 und 150 mg/dl (6,7, 7,2, 7,8 oder 8,3 mmol/l) verwendet werden und bei der Aktivität ein Glukoseanstieg erwartet wird (z. B. nüchterne, hochintensive Aktivität), kann der Glukosezielwert vor Aktivitätsbeginn auf 110 mg/dl (6,1 mmol/l) gesenkt werden und nach der Aktivität wieder auf den üblichen Zielwert zurückgestellt werden (Konsens D). Das System erlaubt die Programmierung von bis zu 8 unterschiedlichen Glukosezielwerten pro Tag und bietet somit eine gewisse Flexibilität. Für Schulkinder kann dies beispielsweise genutzt werden, um 1–2 h vor dem regulären Sportunterricht am Nachmittag einen höheren Zielwert einzustellen und diesen bis zum Ende der Aktivität beizubehalten (Konsens D). Ein zusätzlicher Aspekt für medizinisches Fachpersonal ist die potenzielle Wirkung der Funktion *„Gegenläufige Korrektur“* bei der Mahlzeit vor geplanter Aktivität: Ist diese Funktion aktiviert, wird die prandiale Bolusinsulindosis reduziert, wenn der präprandiale Glukosewert unter dem individuellen Zielwert liegt. Wird gleichzeitig eine manuelle Bolusreduktion durch den/die Nutzer:in vorgenommen (z. B. um minus 25–33 %), kann es infolgedessen zu einem Glukoseanstieg kommen, was wiederum eine automatische Insulinabgabe zur Folge hat und das Risiko für aktivitätsbedingte Hypoglykämien erhöht. Derzeit liegen keine Studien vor, die eine gezielte Empfehlung zum Einsatz der Funktion *„Gegenläufige Korrektur“ *im Kontext physischer Aktivität untermauern. Wenn physische Aktivität < 2 h nach einer Mahlzeit geplant ist und ein Glukoseabfall zu erwarten ist, wird allgemein eine Reduktion der prandialen Bolusinsulindosis um 25–33 % empfohlen [24, 26] (C). Eine alternative Strategie besteht darin, die Funktion *„Gegenläufige Korrektur“* zu deaktivieren, wenn eine manuelle Bolusreduktion vor der Aktivität erfolgt (Konsens D).

Für die Mahlzeit vor Beginn der Aktivität kann die Bolusinsulindosis im Omnipod 5 auf 2 Arten reduziert werden:

1. durch Eingabe einer geringeren Kohlenhydratmenge als tatsächlich konsumiert wird, oder
2. durch manuelle Reduktion der empfohlenen Bolusinsulindosis [9].

Aktuell liegen keine publizierten Studien vor, die den zeitlichen Abstand oder die optimale Höhe der prandialen Bolusinsulinreduktion bei Kindern oder Erwachsenen mit T1DM unter Nutzung des Omnipod 5 evaluieren. Bei Nutzung des Omnipod 5-Systems empfehlen wir, basierend auf Erfahrung, den Glukosezielwert individuell an die erwartete glykämische Reaktion auf physische Aktivität anzupassen (Konsens D). Empfehlungen zum Glukosemanagement bei physischer Aktivität und Sport mit dem Omnipod 5 finden sich in Abb. 4.

## Medtronic MiniMed 780G

Das MiniMed 780G-System (Medtronic MiniMed, Inc., Northridge, California, USA) nutzt die SmartGuard-Technologie und ermöglicht die Einstellung von Glukosezielwerten von 100 mg/dl (5,5 mmol/l), 110 mg/dl (6,1 mmol/l) und 120 mg/dl (6,7 mmol/l). Ein Glukosezielwert von 150 mg/dl (8,3 mmol/l) wird als Sportmodus („*temp. SG-Ziel“)* bezeichnet und kann aktiviert werden, indem man das „*temp. SG-Ziel“* aktiviert. Eine zentrale Sicherheitsfunktion dieses Modus besteht darin, automatische Korrekturboli zu unterbinden, wenn die Glukosekonzentration aufgrund der Aufnahme von Kohlenhydraten unmittelbar vor oder während physischer Aktivität ansteigt. Ohne diese Funktion würde das IOB während der Aktivität signifikant zunehmen, was ein erhöhtes Risiko für wiederkehrende Hypoglykämien zur Folge hätte. Der Auto-Korrekturbolus wird – sofern aktiviert – automatisch abgegeben, wenn der Algorithmus die maximale Basalinsulinrate erreicht, der Sensorglukosewert über 120 mg/dl (6,7 mmol/l) liegt und der berechnete Korrekturbolus mehr als 10 % der maximalen Insulinmenge beträgt. Die SmartGuard-Technologie berücksichtigt bei der Bolusinsulinberechnung für Kohlenhydrate sowohl den aktuellen Glukosewert als auch das gesamten IOB. Die angezeigte IOB (als „Akt. Insulin“ dargestellt) umfasst alle Bolusinsulingaben einschließlich Mahlzeitenboli, manuelle Korrekturboli sowie automatische Korrekturen. Die Basalrate, unabhängig davon, ob es aus einem voreingestellten Profil oder über die SmartGuard-Funktion abgegeben wird, zählt nicht zum IOB. Die Höhe des angezeigten IOB ist abhängig von der individuell einstellbaren Wirkdauer des Insulins (zwischen 2 und 8 h). Der IOB fließt zudem in die Berechnung manueller und automatischer Korrekturboli ein.

## Evidenz zum Glukosemanagement bei physischer Aktivität und Sport mit dem MiniMed 780G-System

Das Medtronic MiniMed-System ist für den Einsatz während physischer Aktivität und Sport bei Menschen mit T1DM geeignet und zählt zu den AID-Systemen mit der umfangreichsten Publikationslage zum Thema Bewegung [8]. In einer Studie mit 10 Erwachsenen mit T1DM zeigte sich, dass der Wechsel vom manuellen Modus zum Automodus im MiniMed 780G-System keinen signifikanten Einfluss auf die Glukosewerte während oder nach einer 45-minütigen moderat-intensiven Belastung hatte [48]. In einer klinischen Pilotstudie bei Erwachsenen mit eingeschränkter Hypoglykämiewahrnehmung von Lee et al. lag die Zeit im Zielbereich (70–180 mg/dl; 3,9–10,0 mmol/l) bei 100 % während 45 min hoch- bzw. moderat-intensiver Trainingseinheiten, wenn das „*temp. SG-Ziel“* beim MiniMed 670G-System 2 h vor Belastungsbeginn aktiviert wurde [25]. McCarthy et al. zeigten, dass die Erhöhung des Glukosezielwertes zu Beginn oder 45 min vor der Aktivität weniger effektiv war zur Vermeidung von Hypoglykämien als eine Erhöhung des Glukosezielwertes 90 min vor der Aktivität in Kombination mit einer Mahlzeitenbolusreduktion [24]. In derselben Studie zeigte sich, dass sich durch eine Reduktion der prandialen Bolusinsulindosis um 25 % bei Mahlzeiten bis zu 90 min vor Aktivität die Glukosewerte während körperlicher Belastung optimieren lassen [24]. In einer Studie mit Jugendlichen mit T1DM war physische Aktivität sicher – unabhängig davon, ob schnelleres Insulin aspart oder Standardinsulin aspart verwendet wurde – wenn das „*temp. SG-Ziel“* mindestens 1 h vor der Aktivität aktiviert wurde [49].

## Empfehlungen zum Glukosemanagement bei physischer Aktivität und Sport mit dem MiniMed 780G-System

**Abb. 5:**
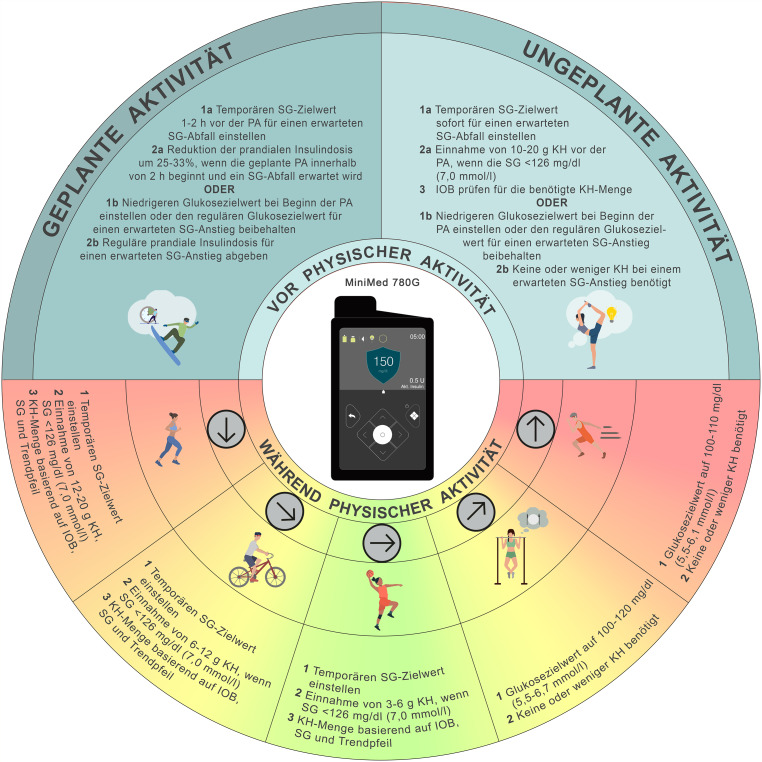
Empfehlungen zur Anwendung des MiniMed 780G-Systems zur Steuerung der Glukosewerte während physischer Aktivität und Sport (*PA*): eine Unterbrechung der Insulinzufuhr mit oder ohne Abkoppelung über einen Zeitraum von bis zu 120 min kann bei bestimmten Aktivitäten (z. B. Schwimmen, Tauchen, Kontaktsport oder in Wettkampfsituationen) erforderlich sein. Für die Mehrheit der Aktivitäten wird dies jedoch nicht empfohlen. *SG* Sensor-Glukosewert, *KH* Kohlenhydrate, *IOB* Insulin On Board

Wenn ein Glukoseabfall zu erwarten ist, kann das *„temp. SG-Ziel“* 1–2 h vor Aktivitätsbeginn aktiviert werden [24, 25]. Die Funktion wird automatisch nach der eingestellten Dauer beendet. Bei Bedarf bzw. falls sinkende Werte nach der Aktivität erwartet werden, kann das *„temp. SG-Ziel“ *für einen weiteren Zeitraum aktiviert bleiben [37, 38]. Bei zu erwartendem Glukoseabfall kann das MiniMed 780G-System im Automodus belassen und mit zusätzlicher Aktivierung des *„temp. SG-Ziel“* sowie mit Reduktion der prandialen Bolusinsulindosis um 25–33 % das Auftreten von Hypoglykämien im Rahmen von körperlicher Aktivität und Sport vermieden werden [26, 48]. Wird eine Reduktion der prandialen Bolusinsulindosis geplant, sollte diese immer in Kombination mit dem *„temp. SG-Ziel“ *erfolgen, da diese Funktion die Autokorrekturfunktion des Algorithmus deaktiviert. Bei ungeplanter Aktivität mit zu erwartendem Glukoseabfall ist in der Regel eine Kohlenhydratzufuhr von 10–20 g erforderlich, insbesondere wenn der Glukosewert zu Beginn < 126 mg/dl (< 7,0 mmol/l) liegt [42] (C). Zudem sollte das *„temp. SG-Ziel“* unmittelbar vor der Kohlenhydratzufuhr aktiviert werden [9] (Konsens D). Wenn hingegen ein Glukoseanstieg erwartet wird, kann ein niedrigerer Zielwert (z. B. 100 mg/dl; 5,5 mmol/l) zweckmäßig sein, um die automatisierte Insulinabgabe zu erhöhen. Bei der Wahl des Zielwertes sollte man sich jedoch an individuellen Glukosereaktionen orientieren, die unter anderem durch Bewegungsart, Tageszeit, Kohlenhydratstrategie, Menstruationsphase oder andere Faktoren individuell beeinflusst werden können (Konsens D). Bei Nutzung des MiniMed 780G-Systems empfehlen wir, basierend auf Erfahrung, den Glukosezielwert individuell an die erwartete glykämische Reaktion auf physische Aktivität anzupassen [50–52] (Konsens D). Zusätzlich besteht die Möglichkeit, die Autokorrekturfunktion auszuschalten oder den Algorithmus abzustellen und lediglich die prädiktive Hypoabstellung zu nutzen (Abb. 5).

## Tandem t:slim X2 mit Control-IQ-Technologie

Die Insulinpumpe t:slim X2 (Tandem Diabetes Care, Inc., San Diego, California, USA) mit Control-IQ-Technologie prognostiziert Glukosewerte 30 min im Voraus und passt die Insulinabgabe entsprechend an. Dabei kann 1‑mal pro Stunde ein automatischer Korrekturbolus abgegeben werden, sofern notwendig. Die Einstellungen im „*persönlichen Profil“* sehen einen Standardglukosezielwert von 110 mg/dl (6,1 mmol/l) vor. Das System strebt jedoch eine Glukosekontrolle im Bereich von 112–160 mg/dl (6,2–8,9 mmol/l) an. Für physische Aktivität und Sport kann ein erhöhter Zielbereich von 140–160 mg/dl (7,8–8,9 mmol/l) aktiviert werden („*Bewegung“*). Im „*Schlaf*“ Modus wird ein engerer Zielbereich zwischen 113–120 mg/dl (6,3–6,7 mmol/l) verwendet. Dieser Modus basiert auf einer etwas aggressiveren Anpassung der basalen Insulingabe und gibt keine automatischen Korrekturboli ab. Obwohl der „*Schlaf*“ Modus ursprünglich für das nächtliche Glukosemanagement konzipiert wurde, können Benutzer:innen Schlafzeiträume zu beliebigen Tageszeiten definieren, um von einer engeren Zielwerteinstellung zu profitieren. Nicht modifizierbare Parameter im Control-IQ-System sind die Insulinwirkdauer und der Glukosezielwert [53]. Ist Control-IQ aktiviert, umfasst das angezeigte IOB (aktives Insulin) sämtliche basalen Insulinabgaben oberhalb oder unterhalb der programmierten Basalrate sowie alle Bolusgaben (Wirkdauer: fest auf 5 h eingestellt). Es können bis zu 6 persönliche Profile erstellt werden, in denen Basalraten, Kohlenhydratfaktoren und Insulinsensitivitätsfaktoren individuell eingestellt werden können – z. B. für unterschiedliche Arten physischer Aktivität und Sport. Ein automatischer Korrekturbolus (max. 60 % der berechneten Korrekturdosis) wird 1‑mal pro Stunde abgegeben, wenn folgende Bedingungen erfüllt sind: Der prognostizierte Glukosewert in 30 min liegt über 180 mg/dl (10,0 mmol/l), das System befindet sich nicht im *„Schlaf“*-Modus, und es wurde in den letzten 60 min kein manueller Bolus abgegeben oder abgebrochen [54]. Das neue Tandem Mobi-System nutzt ebenfalls den Control-IQ-Algorithmus, bietet jedoch eine kompaktere Bauform und kann vollständig über eine Smartphone-App gesteuert werden (inkl. optionalem Bolus-Knopf direkt am Gerät).

## Evidenz zum Glukosemanagement bei physischer Aktivität und Sport mit dem t:slim X2 Control-IQ-System

Das Control-IQ-System von Tandem war das erste AID-System, das bei Kindern und Jugendlichen (6 bis 18 Jahre) mit T1DM im ambulanten Bewegungskontext (z. B. Skisport) getestet wurde [55–57]. In einer aktuellen Untersuchung von Mameli et al. wurde bei Jugendlichen (9 bis 18 Jahre) die Zeit im Zielbereich (70–180 mg/dl; 3,9–10,0 mmol/l) während 2 h geplanter Aktivität (90 min nach dem Mittagessen) erhoben [58]. In 2 Gruppen (Ausdauer gefolgt von Kraft und umgekehrt) wurde der Modus „*Bewegung“ *90 min vor der Aktivität bis zum Abendessen aktiviert und die prandiale Bolusinsulindosis um 50 % reduziert. Die Zeit im Zielbereich lag bei 50,4 % und bei 39,6 % (*p* = 0,39).

## Empfehlungen zum Glukosemanagement bei physischer Aktivität und Sport mit dem t:slim X2 Control-IQ-System

**Abb. 6:**
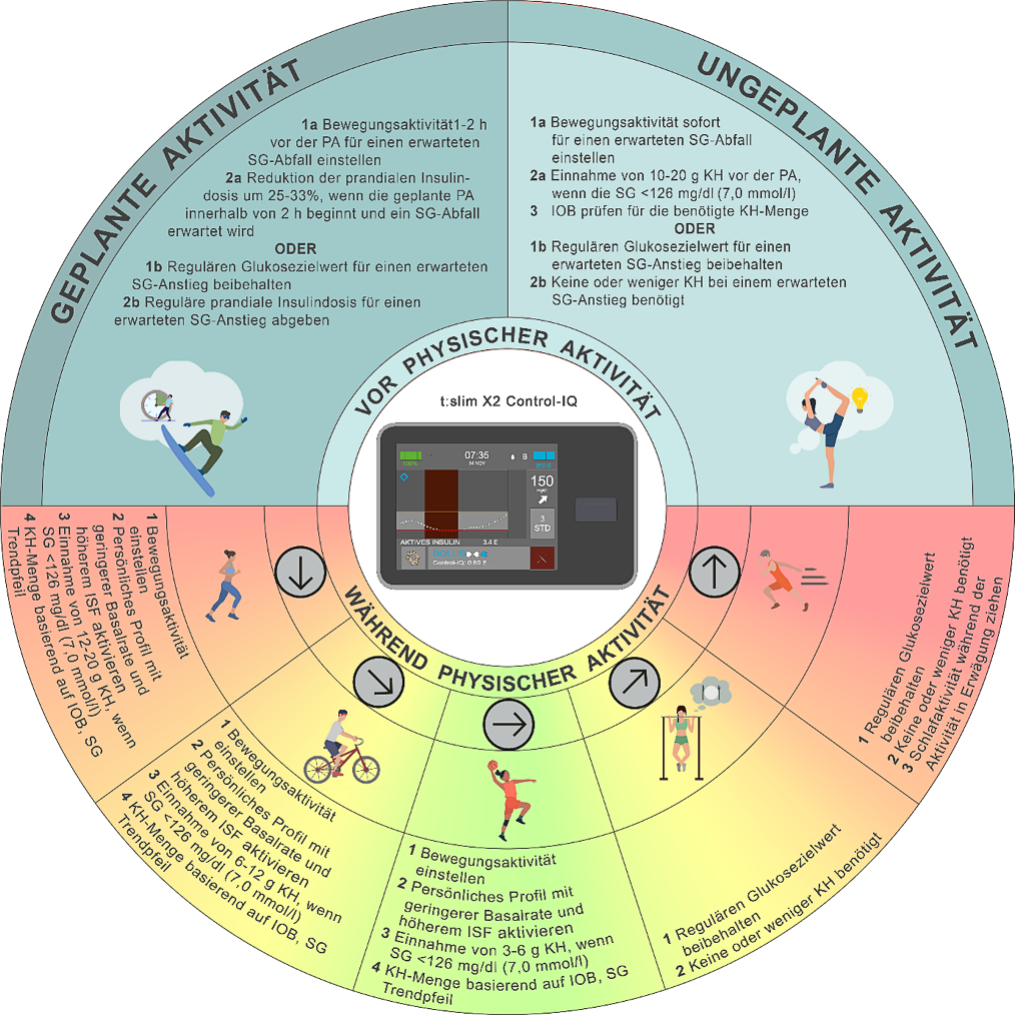
Empfehlungen zur Anwendung des t:slim X2 Control-IQ-Systems zur Steuerung der Glukosewerte während physischer Aktivität und Sport (*PA*): Eine Unterbrechung der Insulinzufuhr mit oder ohne Abkoppelung über einen Zeitraum von bis zu 120 min kann bei bestimmten Aktivitäten (z. B. Schwimmen, Tauchen, Kontaktsport oder in Wettkampfsituationen) erforderlich sein. Für die Mehrheit der Aktivitäten wird dies jedoch nicht empfohlen. *SG* Sensor-Glukosewert, *KH* Kohlenhydrate, *IOB* Insulin On Board, *ISF* Insulinsensitivitätsfaktor

Physische Aktivität und Sport können dem System ankündigt werden, indem der Modus *„Bewegung“* aktiviert wird (Zielbereich 140–160 mg/dl bzw. 7,8–8,9 mmol/l) (s. Abb. 6). Sinkt die prognostizierte Glukose 30 min später unter 140 mg/dl (7,8 mmol/l), reduziert das System die Insulinzufuhr. Liegt der prognostizierte Wert über 160 mg/dl (8,9 mmol/l), erhöht das System die Insulinzufuhr. Bei erwarteten Werten über 180 mg/dl (10,0 mmol/l) erfolgt ein automatischer Korrekturbolus (60 % der regulären Korrekturdosis), auch im Modus *„Bewegung“* [58]. Dies kann das Risiko einer Hypoglykämie während der Aktivität erhöhen, insbesondere bei vorab konsumierten, nicht angekündigten Kohlenhydraten. Kein automatischer Korrekturbolus erfolgt innerhalb von 60 min nach einer beliebigen (auch abgebrochenen) Bolusgabe. Mit der Softwareversion 7.7 (ab Januar 2024 in einigen Ländern verfügbar) kann der Modus *„Bewegung“* für eine Dauer von 30 min bis zu 8 h aktiviert werden. Bei älteren Versionen muss der Modus manuell deaktiviert werden. Empfohlen wird, den Modus *„Bewegung*“ 1–2 h vor Beginn der Aktivität bis zum Ende der sportlichen Bewegung zu aktivieren, um das IOB zu senken und das Hypoglykämierisiko zu reduzieren [25] (Konsens D). Für Personen, die regelmäßig körperlich aktiv sind, kann es hilfreich sein, separate persönliche Glukoseprofile zu programmieren – z. B. für besonders aktive Tage oder für längere Belastungsphasen [9] (Konsens D). Wenn während der Aktivität Kohlenhydrate zugeführt werden (z. B. zur Hypoglykämiebehandlung), kann der resultierende Glukoseanstieg auch im Modus *„Bewegung“* eine Insulinzufuhr auslösen. Daher sollte – wie auch bei anderen AID-Systemen mit erhöhtem Zielwert für Bewegung – mit der zusätzlichen Zufuhr von Kohlenhydraten (frühere Sport BE oder Sport KE) vor und während der Aktivität Zurückhaltung gezeigt werden. Basierend auf Konsens D, kann alternativ ein kleiner manuell verabreichter Bolus (Mindestmenge: 0,05 E) kurz vor Aktivitätsbeginn erwogen werden. Dies deaktiviert die „Auto-Bolus“-Funktion für die nächsten 60 min, selbst wenn ein Glukoseanstieg durch Kohlenhydratkonsum erfolgen sollte. Alternativ kann ein persönliches Profil mit sehr hohem Insulinsensitivitätsfaktor programmiert werden (> 200 mg/dl/IE), um die automatischen Korrekturinsulindosen möglichst gering zu halten. Zudem kann für körperliche Aktivität und Sport ein spezifisches Profil programmiert werden und dementsprechend benannt werden (z. B. Ausdauer‑, Kraft- oder Intervalltraining), das sich durch eine angepasste Basalrate sowie Mahlzeitenfaktoren und einen angepassten Korrekturfaktor im Sinne einer angepassten Insulinsensitivität (ISF) auszeichnet. Für Szenarien, bei denen ein Glukoseanstieg zu erwarten ist, z. B. bei hochintensiven Sprintbelastungen, bedarf es weiterer Untersuchungen und Studien (Konsens D). Bei Nutzung des Tandem t:slim X2-Systems empfehlen wir, basierend auf Erfahrung, den Glukosezielwert individuell an die erwartete glykämische Reaktion auf physische Aktivität anzupassen (Konsens D). Empfehlungen zur Glukosekontrolle mit dem t:slim X2 Control-IQ finden sich in Abb. 6.

## Weitere Aspekte in Zusammenhang mit physischer Aktivität und Sport

Derzeit gibt es nur wenige evidenzbasierte Empfehlungen zu „Extremsituationen“ zum Glukosemanagement bei physischer Aktivität und Sport, besonders im Zusammenhang mit der Nutzung von AID-Technologien. Um diesen spezifischen Situationen besser gerecht zu werden, bietet die folgende Übersicht besondere Konstellationen, relevante Aspekte und mögliche Strategien für Menschen mit T1DM bei der Nutzung von AID-Systemen bei körperlicher Aktivität und Sport (Konsens D):

### Lang andauernde schwere körperliche Belastungen

(z. B. Ultramarathon, Ironman, Triathlon, Radrennen, Trekking/Wandern)

- *Aspekte*: niedrige Insulinzufuhr, erhöhtes Risiko für vermehrte Produktion von Ketonkörpern, Risiko für Hypo- und Hyperglykämien
- *Strategien*:
  - Regelmäßige Kohlenhydratzufuhr während der Belastung
  - Ausreichende Flüssigkeitszufuhr
  - Kein dauerhaft stark erhöhter Glukosezielwert, um Insulinmangel zu vermeiden
  - Keine großen Snacks ohne Bolusinsulinabgabe, da dies die automatisierte Insulinzufuhr signifikant erhöht und dadurch das Hypoglykämierisiko erhöht werden kann
  - Engmaschige Kontrolle der Sensorglukosewerte; ggf. höhere Hypo- und Hyperglykämie-Alarmschwellen einstellen

### Längerfristige Pumpenunterbrechung (> 120 min)

(z. B. bei Kontakt- oder Wassersport)

- *Aspekte*: reduzierte Insulinzufuhr, Risiko für Hyperglykämien, Risiko für Ketonkörperproduktion und Ketoazidose
- *Strategien*:
  - AID-System abschalten, um reduzierte Insulinabgabe im Algorithmus zu dokumentieren
  - Pumpe stündlich verbinden und wieder anschalten, um kleine Insulinboli zur Insulinzufuhr abzugeben
  - Alternativ: alle 60 min beispielsweise ~20–50 % der üblichen Basalrateinsulinmenge pro Stunde manuell mittels Pumpe (oder alternativ via Insulinpen) injizieren
  - Bei Bedarf vor der geplanten Aktivität in den manuellen Modus mit reduzierter Basalrate wechseln und gegebenenfalls mit Insulinpentherapie den Basalratenverlust kompensieren

### Wettkampfstress

(z. B. Fußballspiel, Tennismatch)

- *Aspekte*: stressinduzierte Hyperglykämie, Risiko für verzögerte Hypoglykämie
- *Strategien*:
  - IOB vor dem Wettkampf kontrollieren, da AID-Systeme bei stressbedingter Hyperglykämie möglicherweise mehr Insulin abgeben
  - Ausreichende Flüssigkeitszufuhr
  - Kein erhöhter Glukosezielwert 1–2 h vor dem Wettkampf (erhöhten Glukosezielwert erst direkt vor der Belastung aktivieren)
  - Bei Werten > 270 mg/dl (> 15,0 mmol/l): manuelle Teilkorrektur (z. B. 50 % der üblichen Korrekturdosis) erwägen und zusätzlich die Blut- oder Gewebsketonkonzentration prüfen

### Wasserbasierte Aktivitäten

(z. B. Schwimmen, Surfen, Schnorcheln, Tauchen)

- *Aspekte*: eingeschränkte oder fehlende Kommunikation zwischen CGM und AID-System, Risiko für Hypoglykämie
- *Strategien*:
  - Pumpe abnehmen beim Tauchen
  - Hohes IOB zu Beginn vermeiden
  - AID-System ggf. ablegen und ausschalten, Pumpe stündlich anlegen, wieder anschalten, um kleine Insulinboli zur Insulinzufuhr abzugeben
  - Schnell wirksame Kohlenhydrate in geeigneter Form mitführen

### Kontaktsportarten

(z. B. Ringen, Judo, Boxen, Taekwondo)

- *Aspekte*: mechanische Belastung und mögliche Beschädigung der Geräte, Stressreaktion mit Hyperglykämie
- *Strategien*:
  - Geräteplatzierung für besseren Schutz anpassen
  - CGM und Pumpe mit Fixierpflaster sichern
  - AID-System ggf. ablegen und ausschalten, Pumpe stündlich anlegen, wieder anschalten, um kleine Insulinboli zur Insulinzufuhr abzugeben
  - Kein erhöhter Zielwert vor der Aktivität, wenn ein Risiko für Hyperglykämie besteht

### Hohe Umgebungstemperaturen

(z. B. Wandern bei Hitze)

- *Aspekte*: vermehrtes Schwitzen, bessere Insulinresorption, CGM-Ungenauigkeit, Risiko für Hypoglykämie
- *Strategien*:
  - Ausreichende Flüssigkeitszufuhr
  - Im Zweifel blutige Glukosemessungen durchführen
  - Glukosezielwert 1–2 h vor Beginn der Aktivität erhöhen
  - Mahlzeiten‑/Snackbolus um 25–33 % reduzieren
  - CGM und Pumpe mit Fixierungen sichern

### Niedrige Umgebungstemperaturen

(z. B. Skifahren, Langlaufen, Skitouren)

- *Aspekte*: zu tiefe Temperaturen für Insulinlösung (einfrieren, ausflocken, verfärben), Frieren von flüssigem Glukosegel, Stressreaktionen mit Hyperglykämie, Signalverlust zwischen Pumpe und Sensor
- *Strategien*:
  - Insulin und Kohlenhydrate nah am Körper tragen (Körperwärme)
  - Nicht gefrierempfindliche Hypoglykämiebehandlung mitführen (z. B. Traubenzucker)
  - Glukosemessgeräte ggf. vor der Glukosemessung in warme Umgebung bringen (z. B. Berghütte)
  - Sensorglukosewerte engmaschig im Blick behalten (z. B.: alle 10–15 min)

### Hochgebirgslagen

(z. B. über 5000 m Höhe)

- *Aspekte*: Hypoxie, erhöhte Insulinresistenz, hormonelle Gegenregulation, CGM-Messungenauigkeit
- *Strategien*:
  - Blutglukosemessgerät anstelle von CGM verwenden
  - Ausreichend Flüssigkeit zuführen
  - Begrenzte Evidenz zur AID-Nutzung in großen Höhen
  - Bei akuter Höhenexposition mögliche Insulinresistenz einplanen
  - Ab 5000 m: möglicher Kortisolanstieg → erhöhtes Hyperglykämierisiko
  - Durchführung von Blut- oder Gewebsketonmessungen

## Resümee

In dieser gemeinsamen Leitlinie der DDG und ÖDG werden sowohl allgemeine Strategien als auch systemspezifische Empfehlungen zur Anwendung von AID-Systemen, welche in Deutschland und Österreich Verwendung finden, vorgestellt. Diese Leitlinie soll nicht nur Fachkräfte im Gesundheitswesen, sondern auch Menschen mit T1DM bei der effektiveren Nutzung dieser innovativen Technologien im Kontext geplanter und ungeplanter physischer Aktivität und Sport unterstützen. Es wird betont, dass diese Empfehlungen als Orientierungshilfe zu verstehen sind und dass individuelle Glukoseschwankungen bei physischer Aktivität und Sport gemeinsam mit dem betreuenden medizinischen Team reflektiert und diskutiert werden sollten. In der täglichen Praxis können weitere individuelle Anpassungen und Feinabstimmungen erforderlich sein, da auch nach Implementierung entsprechender Maßnahmen mit unvorhersehbaren glykämischen Reaktionen gerechnet werden muss. Trotz individueller Unterschiede in der glykämischen Reaktion auf verschiedene Aktivitätsformen hoffen wir, dass diese evidenzbasierten Handlungsempfehlungen dazu beitragen, das selbstständige Glukosemanagement bei physischer Aktivität und Sport für Menschen mit Diabetes zu optimieren und zu erleichtern.

## Interessenkonflikt

O. Moser gibt folgende Offenlegungen an: Unterstützung für klinische Studien erfolgte durch die Sêr Cymru II COFUND Fellowship/Europäische Union, Novo Nordisk A/S, Novo Nordisk AT, Abbott Diabetes Care, Sanofi, Dexcom, Team Novo Nordisk, SAIL, Maisel’s Brauerei, Medtronic AT, EFSD/EASD, Falke, BISp, perfood, Ypsomed sowie Sinocare. Vortragshonorare wurden von Medtronic AT, Medtronic International, Eli Lilly, Novo Nordisk, Sanofi, TAD Pharma, ADA, Diatec, dem Berufsverband Deutscher Internist*innen, Dexcom, AstraZeneca, Ypsomed, Insulet, der Arbeitsgemeinschaft der Diabetologen in Hessen sowie Abbott erhalten. Reisekostenunterstützung für Kongressteilnahmen wurde durch Novo Nordisk A/S, Novo Nordisk AT, Novo Nordisk UK, Medtronic AT, Sanofi, EASD, ÖDG und DDG gewährt. Mitgliedschaften in wissenschaftlichen Beiräten bestehen bei Sanofi, TAD Pharma, Dexcom, perfood sowie Medtronic. U. Becker gibt folgende Offenlegungen an: Honorare für Vorträge von AstraZeneca, Boehringer Ingelheim und Berlin-Chemie; Kongressteilnahme und Reisekostenunterstützung: AstraZeneca. L. van den Boom gibt folgende Offenlegungen an: Honorare für Vorträge erhalten von Medtronic, Insulet, Dexcom, Sanofi, E. Lilly. C. Brinkmann gibt folgende Offenlegungen an: Honorare für Vorträge von E. Lilly, Abbott Diabetes Care und Novo Nordisk. Forschungsunterstützung durch Abbott Diabetes Care. Mitglied im Beraterboard bei Abbott Diabetes Care. T. Danne gibt folgende Offenlegungen an: Er ist der Chief Medical Officer von Breakthrough T1D und hat erklärt, dass diese Institution Zahlungen oder Honorare von AstraZeneca, Abbott, Biomedia, Bayer, Boehringer Ingelheim, Dexcom, Eli Lilly, Insulet, Medtronic, Menarini, Novo Nordisk, Roche, Sanofi, Vitalaire und Ypsomed erhalten hat. T. D. hat an einem Data Safety Monitoring Board oder Advisory Board für Medtronic und Eli Lilly teilgenommen und erklärt, dass er der Mitbegründer von DreaMed Ltd. ist. E. Fröhlich-Reiterer gibt folgende Offenlegungen an: Forschungsunterstützung und Honorare für Vorträge von Sanofi, E. Lilly, Medtronic, Merck, Sandoz. B. Gehr gibt folgende Offenlegungen an: Vortrags- und Beratungshonorare von Abbott, Ascensia, Dexcom, Diabeloop, IME-DC, Insulet, Lilly, Novartis, Novo Nordisk, Medtronic, Roche, Vitalaire. A.-T. Kietaibl gibt folgende Offenlegungen an: Honorare von Sanofi Aventis GmbH und Roche Diabetes Care Austria GmbH erhalten. Kongressunterstützung durch Novo Nordisk, Eli Lilly, GmbH, Boehringer Ingelheim und ÖDG. G. Koehler gibt folgende Offenlegungen an: Honorare für Vorträge, Advisory Boards oder Reisekostenunterstützung von: Amgen, AstraZeneca, Boehringer Ingelheim, Daiichi Sankyo, Eli Lilly, Novo Nordisk AT, Dexcom, Roche, Sanofi Aventis, Austro Control. S. Kress gibt folgende Offenlegungen an: Honorare für Vorträge erhalten von Abbott, AstraZeneca, Bayer Vital, Boehringer Ingelheim, Eli Lilly, Merck Sharp & Dohme, Novo Nordisk, Sanofi. J.K. Mader gibt folgende Offenlegungen an: Advisory Boards von Abbott Diabetes Care, Becton Dickinson, Biomea Fusion, Dexcom, Eli Lilly, Embecta, Insulet, Medtronic, myLife, Novo Nordisk A/S, Pharmasens, Roche Diabetes Care, Sanofi-Aventis, Tandem und Viatris; Honorare für Vortragstätigkeit: A. Menarini Diagnostics, Abbott Diabetes Care, Dexcom, Eli Lilly, Medtrust, MSD, Novo Nordisk A/S, Roche Diabetes Care, Sanofi, Viatris und Ypsomed. Gesellschafterin: decide Clinical Software GmbH und elyte Diagnostics. B. Rami-Merhar gibt folgende Offenlegungen an: Honorare für Vorträge oder Advisory Boards erhalten von Eli Lilly, Sanofi, Medtronic, Abbott Diabetes Care, Insulet. D. Pesta gibt an, keine Offenlegungen zu haben. S. Sanfilippo gibt folgende Offenlegungen an: Unterstützung der Promotion durch ein Promotionsstipendium der Studienstiftung des deutschen Volkes. Unterstützung für klinische Studien erfolgte durch Ypsomed. Reisekostenunterstützung für Kongressteilnahmen wurde von Ypsomed gewährt. I. Schütz-Fuhrmann gibt folgende Offenlegungen an: Honorare für Vortragstätigkeit: Abbott Diabetes Care, Roche Diabetes Care, Dexcom. H. Sourij gibt folgende Offenlegungen an: Advisory Boards von Amgen, Amarin, Boehringer Ingelheim, Bayer, Eli Lilly, Daiichi Sankyo, Novartis, Novo Nordisk, Sanofi-Aventis; Honorare für Vortragstätigkeit: Amgen, Amarin, Boehringer Ingelheim, Bayer, Eli Lilly, Daiichi Sankyo, Novartis, Novo Nordisk, Sanofi-Aventis. M. Tauschmann gibt folgende Offenlegungen an: Vortragshonorare von Medtronic AT, Eli Lilly, Sanofi, Diatec und Ypsomed erhalten zu haben. Reisekostenunterstützung für Kongressteilnahmen wurde durch KelCon GmbH, Ypsomed und ÖDG gewährt. Mitgliedschaften in wissenschaftlichen Beiräten bestehen bei Sanofi, Abbott. U. Thurm gibt folgende Offenlegungen an: Vortragshonorare von Abbott, AstraZeneca, Dexcom, Diashop, Glooko, Lilly Deutschland, Medtrix Verlag, Medtronic, Novo Nordisk, Roche Diabetes Care, Ypsomed, Sanofi-Aventis. G. Treiber gibt folgende Offenlegungen an: Honorare für Vorträge und Reisekostenunterstützung für Kongressteilnahme erhalten von Medtronic AT, Sanofi, E. Lilly, Novo Nordisk, Bayer Austria, ÖDG, Österreichische Akademie der Ärzte. S. Hofer gibt folgende Offenlegungen an: Forschungsunterstützung und Honorare für Vorträge erhalten von Ypsomed, Sanofi, E. Lilly.
